# Thyroid Bethesda Category AUS/FLUS in Our Microscopes: Three-Year-Experience and Cyto-Histological Correlation

**DOI:** 10.3390/cancers11111670

**Published:** 2019-10-28

**Authors:** Roope Huhtamella, Ivana Kholová

**Affiliations:** 1 Department of Pathology, Fimlab Laboratories, 33520 Tampere, Finland; 2 Faculty of Medicine and Health Technology, Tampere University, 33520 Tampere, Finland

**Keywords:** TBSRTC, Bethesda System reporting, thyroid, FNA, AUS/FLUS, atypia

## Abstract

The Bethesda System for Reporting Thyroid Cytopathology (TBSRTC) introduced a new category: Atypia of Undetermined Significance/Follicular Lesion of Undetermined Significance (AUS/FLUS) comprising of heterogenous lesions with a lesser degree of atypia. Its routine use is a bit controversial. The study cohort included AUS/FLUS thyroid cytopathological diagnoses signed out at Fimlab Laboratories from the period of 1 October 2013 to 31 December 2016. We analyzed all the AUS/FLUS cases, their cytology subclassification, and their cyto-histological correlation, when available. In total, there were 331 AUS/FLUS cases from 252 patients. The mean age was 59.8 years and there were 196 females and 56 males. Repeated AUS/FLUS was diagnosed in 75 (29.8%) cases. Out of 252 patients, 118 (46.8%) were operated on. Sixty-eight were operated on after the first AUS/FLUS diagnosis, 46 after 2 AUS/FLUS diagnoses, and 4 after 3 AUS/FLUS diagnoses. In total, there were 37 (14.7%) malignancies and 40 benign tumors. The risk of malignancy for AUS/FLUS (14.7%) is in agreement with the original TBSRTC risk of malignancy. The risk of neoplasia was 30.6% in our series.

## 1. Introduction

Thyroid fine-needle aspiration (FNA) is a key method for evaluating thyroid nodules. The Bethesda System for Reporting Thyroid Cytopathology (TBSRTC) [1], which has been updated via a recently published second edition, is widely adopted in thyroid FNA reporting all around the world [2,3,4,5,6,7,8,9,10]. TBSRTC introduced a new category: Atypia of Undetermined Significance/Follicular Lesion of Undetermined Significance (AUS/FLUS) comprising of lesions with a lesser degree of atypia. The heterogeneity of the morphological features, both cytological and architectural, encourages the subclassification of the lesions. The suggested scenarios from the first edition were further elaborated in the second edition subclassification [1,2]. 

AUS/FLUS routine use is variable. In a survey from the College of American Pathologists on trends in thyroid FNA, it was the second most common diagnosis after benign cases, comprising 8.6% of cases in 2016 in comparison to 7.7% in 2011 [11]. Accordingly, the frequency of AUS/FLUS category increased from 4% in the pre-TBSRTC period to 8% in the post-TBSRTC period, even up to a 12% peak in the last analyzed year in an institutional study [12]. In the larger analyses, the AUS/FLUS diagnosis ranged from 0.8–28%, with marked interobserver and intraobserver variations based on FNA aspirator, specimen preparation, and staining method [13,14,15].

The risk of malignancy (ROM) and risk of neoplasia (RON) also differ in various studies, from 6–97% (ROM) and from 6–56% (RON), despite the aim to serve as quality control guides [14,15]. Furthermore, RON is inconsistently defined in different series [14,15,16].

In the present study, we show our institutional analysis of AUS/FLUS cases with subclassification, cyto-histological correlation, ROM, and RON.

## 2. Results

A search yielded 1698 thyroid FNAs performed between 1 October 2013 to 31 December 2016 and of them 331 (19.5%) FNAs were diagnosed as AUS/FLUS category. After implementing exclusion criteria, 252 patients and 331 AUS/FLUS FNAs remained in the cohort. The mean age of the patients ± standard deviation (SD) was 59.8 ± 15.8 years, with patient ages ranging from 15 to 99 years. There were 196 (77.8%) females and 56 (22.2%) males. In total, 118 (46.8%) cases underwent surgical resection. Seventy-six lobectomies and 42 total thyroidectomies were performed. Average nodule size ± SD was 24 ± 14 mm. The study cohort is summarized in Table 1.

**Figure 1:**
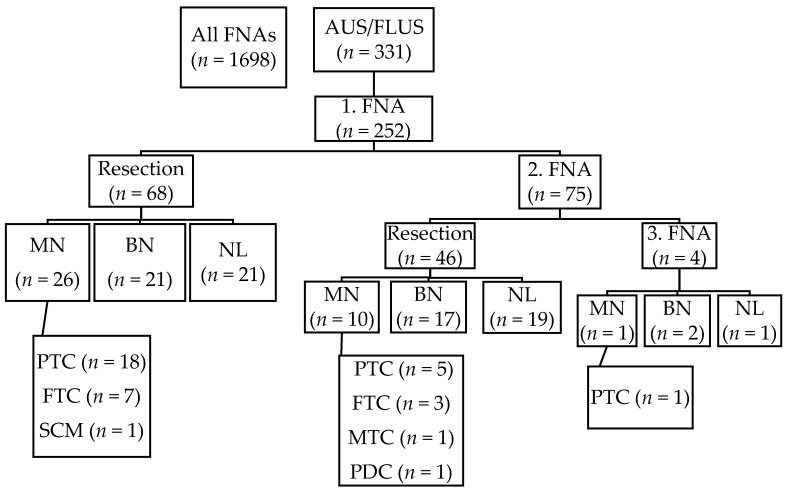
The outcome of AUS/FLUS cases. MN, malignant neoplasms; BN, benign neoplasms; NL, non-neoplastic lesions, PTC, papillary thyroid carcinoma; FTC, follicular thyroid carcinoma; MTC, medullary thyroid carcinoma; PDC, poorly differentiated carcinoma; SCM, squamous cell carcinoma metastasis.

Sixty-eight out of one hundred and seventy-seven patients were operated on after the first AUS/FLUS diagnosis. Seventy-one (28.2%) patients had two consecutive AUS/FLUS results, and out of them, 46 (64.8%) were operated on. Four patients had three consecutive AUS/FLUS diagnoses, and all of them were operated on. The malignant histopathological diagnoses were as follows: 24 (64.9%) papillary carcinomas, 10 (27.0%) follicular carcinomas, one medullary carcinoma, one poorly differentiated carcinoma, and one squamous cell carcinoma metastasis. Only one papillary carcinoma was re-classified as non-invasive follicular neoplasm with papillary-like features (NIFTP). Forty follicular adenomas were diagnosed, and 41 cases had non-neoplastic outcomes (Figure 1). 

Multiple malignant diagnoses were found in three cases: two of them were follicular carcinomas with incidental papillary microcarcinoma, and one case was with two different tumors—poorly differentiated carcinoma in the left lobe and papillary carcinoma (follicular variant) in the right lobe. In addition, a follicular adenoma was found in nine malignant cases: all of them were papillary carcinomas—eight were microcarcinomas and one was a classical, multifocal variant. 

The risk of malignancy (ROM) was 14.7% (lower bound, LB) in the whole cohort and 31.1% (upper bound, UB) in resected cases. Risk of neoplasia (RON) was 30.6% in all cases and 64.7% in resected cases. Further, we subanalyzed ROM in cases with a single AUS/FLUS diagnosis: it was 14.7% for all cases and 38.2% for surgically confirmed cases. RON was 26.6% and 69.1%, respectively. Cases with two consecutive AUS/FLUS diagnoses had 14.1% (UB) and 21.7% (LB) ROM. RON was 38.0% and 58.7%, respectively (Table 2). 

The most common subcategory for AUS/FLUS FNAs was group 1 (follicular neoplasia), with 125 cases (37.8%) and with a ROM of 12.0%; the second most common category was group 6 (nuclear enlargement), with 91 cases (27.5%) and with a ROM of 11.0%; and the third most common was group 4 (focal features suggestive of papillary carcinoma), with 72 cases (21.8%) and with a ROM of 16.7%. Only seven cases belonged to group 5 (atypical cyst-lining cells), with a ROM of 26.6%. All cases were later categorized according the second edition Bethesda: the most common was architectural atypia (37.8%), the second most common was cytological atypia (29.6%), and the third most common was cytological and architectural atypia (21.8%). ROMs were respectively 12.0%, 12.2%, and 16.7%. To complete, 8.5% of cases were categorized as oncocytic cell aspirates, 0.3% as atypical lymphoid cells, and 2.1% as atypia not otherwise specified (NOS) (Table 3).

## 3. Discussion

In the present study of 331 AUS/FLUS cases from 252 patients, 46.8% of cases were histologically correlated. There were 37 (14.7%) malignant tumors and 40 benign tumors in total. Only one papillary carcinoma was re-classified as NIFTP. In the non-neoplastic resections, six cases of thyroiditis and two cases of hyperthyroidism were diagnosed. Those non-neoplastic entities are well recognized for cytomorphological pitfalls [17,18].

A meta-analytic review of 47 studies of 4475 surgically treated AUS/FLUS cases showed 27% (range 23–31) ROM [16]. In the present study, the ROM for all cases (14.7%) is in agreement with original TBSRTC ROM; however, it was 31.1% for the operated cases. Interestingly, ROM was 38.2% in histologically confirmed cases after the first FNA and 21.7% after the second FNA. The influence of repeated FNA on ROM was also studied by Kuru et al., who observed 7% ROM (LB) and 23% ROM (UB) in all cases, but if cases with repeat FNA were evaluated, ROM was 38.6% vs. 15% in cases without repeat FNA [19]. After the first AUS/FLUS with repeated benign FNA diagnosis, ROM was still 18% in excised cases. Two consecutive AUS/FLUS revealed a 50% ROM and AUS/FLUS/malignant cytology revealed a 100% ROM [12]. In contrast, repeated FNA had no influence on ROM in the Ho et al. study (26.3% vs. 26.6%) [20]. Interestingly, in an Asian practice of active surveillance, high ROMs in the indeterminate FNA categories were observed. On average, the Asian ROM was 44%, with a range of 14.3–75%, with only the Japanese results similar to the original TBSRTC values. They concluded that stricter criteria for the papillary carcinoma cytological diagnosis mirror this discrepancy [21]. The most common biases in the ROM analyses are papillary microcarcinomas; surgically treated patients are likely to have other (i.e., clinical or imaging) features of malignancy and tertiary centers with higher percentages of malignancies [20,21,22,23].

Throughout the studies, RON was calculated sparsely. RON was 30.6% in our whole series and 64.7% for operated cases. RON was 69.1% after the first FNA and 58.7% after the second FNA in cases with cyto-histological correlation in our series. Dincer et al. reported a RON of 34.1% in a series of 368 patients with 19.6% surgically verified cases [5]. The literature analysis revealed a RON range of 6–56% [14]. 

The fourth edition of *WHO Classification of Tumours of Endocrine Organs* incorporated several borderline/precursor tumors [24]. NIFTP onset brought along a new pitfall, as nuclear features of papillary carcinoma can be present in the entity with an extremely low malignant potential [24,25,26]. In various studies and analyses, the ROM of AUS/FLUS declined when NIFTP was implemented as non-malignancy [27,28]. Nevertheless, the impact of NIFTP in an Asian cohort was much lower than in Western reports [29]. The influence of NIFTP on ROM is marginal in our analysis due to only one NIFTP case. 

We observed a wide variation of ROM values in cases stratified according to cytomorphological features. Surprisingly, the highest ROM was 28.6% in cases with atypical cyst-lining cells. In atypical cyst-lining cell specimens, there is a pitfall of distinguishing benign reparative cystic changes in goiter vs. cystic papillary carcinoma. Comprehensive cytomorphological analysis of atypical cyst-lining cells in FNA concluded that lack of nuclear crowding, intranuclear pseudoinclusions, and papillary architecture defend benign lesions [30]. 

The second highest ROM was in cases with focal features suggestive of papillary carcinoma, with a ROM of 16.7%. On the other hand, the predominance of oncocytic cells was accompanied by a ROM of only 4.2%. Notably, the ROM of oncocytic cell lesions ranged from 14–70% in 1397 meta-analyzed histologically verified cases [16]. 

After re-categorization in agreement with the second Bethesda system, oncocytic cell predominant aspirates featured a ROM of 3.6%. The cases with both cytological and architectural atypia had a ROM of 16.7%, and cytological atypia had a ROM of only 12.2% and architectural atypia had a ROM of only 12.0%. Statistically, nuclear features such as grooving and irregularity were shown to be significantly related to malignancy [31]. From a clinical point of view, the subclassification and knowledge of the ROM of each subcategory is important. Lesions with features suggestive of papillary carcinoma and both cytological and architectural atypia have a higher ROM than oncocytic cell aspirates. In summary, subclassification is encouraged to enhance communication with other pathologists and clinicians and to facilitate further refinement of the AUS/FLUS category [2].

## 4. Materials and Methods 

A retrospective search was conducted to find all AUS/FLUS cases diagnosed at the Department of Pathology, Fimlab Laboratories, Tampere, Finland, between 1 October 2013 and 31 December 2016. The department serves Pirkanmaa Hospital District area. Cases with one or more AUS/FLUS diagnoses in FNAs were included in the study cohort as well as their cytological or histological follow up when available until 31 December 2017. 

All FNA samples were taken under ultrasound guidance with a 22G needle and were alcohol-fixed and cytospun and Papanicolaou stained. All FNA samples were classified according to the TBSRTC [1,2] into six categories: non-diagnostic, benign, atypia of undermined significance/follicular lesion of undetermined significance (AUS/FLUS), follicular neoplasm/suspicious for follicular neoplasm (FN/SFN), suspicious for malignancy (SM), and malignant. Seventy-five percent of all samples were diagnosed by two senior pathologists who are specialized in thyroid cytopathology. If more than one nodule was sampled or the same nodule was sampled twice in the same examination, the most severe result was included in our analysis. 

Cohort data were collected from pathology reports and included the indication for FNA, nodule size, age, sex, FNA result and subclassification, type of surgical removal, and histopathological diagnosis, when available. Histological verification and other FNAs of the thyroid gland and their outcomes were matched in each case. Neoplasms were classified according to WHO classifications for thyroid tumors [24]. All follicular variants of papillary carcinomas were scrutinized for the possibility of NIFTP diagnosis. Other histological findings were classified as goiter, thyroiditis, and hyperthyroidism. 

We estimated the risk of malignancy (ROM) and neoplasia (RON) by calculating the upper and the lower bounds because only a subset of patients with AUS/FLUS diagnosis had surgical follow-up. Upper bound is calculated by having all resected cases as denominator and malignant or neoplasia cases as numerator. For the lower bound we used the total number of cases as denominator. The correct ROM or RON settles somewhere between these two values [2]. 

Subclassification was done according to the first edition Bethesda classification, which differs from the newer second edition. Our data used eight categories: follicular neoplasia (group 1), predominance of oncocytes (group 2), only oncocytes (group 3), focal features suggestive of papillary carcinoma (group 4), atypical cyst-lining cells (group 5), nuclear enlargement (group 6), atypical lymphoid infiltrate (group 7), and others (group 8). We matched the first edition Bethesda categories to the second edition Bethesda categories as follows: the first group was classified as ‘architectural atypia’, the second and the third groups were combined into the ‘oncocytic cell aspirates’ classification, the fourth group was classified as ‘cytological and architectural atypia’, the fifth and the sixth groups were combined into the ‘cytologic atypia’ classification, the seventh group was classified as ‘lymphoid infiltrate’, and the eighth group was classified as ‘others’. Furthermore, we subanalyzed the ROM in each category. Due to data size, here we used all FNAs as the denominator.

Data and statistical analyses were performed with Microsoft Excel software (Microsoft, Redmond, WA, USA).

This study was approved by the Regional Ethics Committee of Pirkanmaa Hospital District (R13168) and informed consent of each individual was not requested. The study was conducted according to the Declaration of Helsinki. 

## 5. Conclusions

In conclusion, the institutional analysis with cyto-histological correlation showed a 14.7% ROM, which is in agreement with the original TBSRTC. The RON, which has been inconsistently reported in various studies and analyses, was 30.6% in our series. The subclassification pointed out variable ROM of each subcategory. Despite the morphological heterogeneity, AUS/FLUS is a well-defined TBSRTC category with both diagnostic and management criteria. 

## Figures and Tables

**Table 1:** Description of the study cohort. FNA, fine-needle aspiration; AUS, atypia of undetermined significance.

Characteristic	*n*	%
Sex		
Female	196	77.8
Male	56	22.2
Age ± SD (years)	59.8 ± 15.8	
Indication of FNA		
Nodule	130	
Goiter	114	
Other	5	
No data	3	
Nodule size ± SD (mm)	24 ± 14	
≤1 cm	24	18.5
>1–2 cm	41	31.5
>2–4 cm	50	38.5
>4 cm	13	10.0
No data	2	1.5
Operated	118	46.8
Total thyroidectomy	42	35.6
Lobectomy	76	64.4
Reoccurring AUS-FNA	75	29.8

**Table 2:** The risk of malignancy and neoplasia in AUS/FLUS category.

Risk of Malignancy/Neoplasia	Study Cohort		Bethesda Values [1]		Meta-Analysis Straccia et al. [17]
	% of All (Lower Bound)	% of Resected (Upper Bound)	% of All (Lower Bound)	% of Resected (Upper Bound)	% of Resected (95% CI)
Risk of malignancy	14.7	31.1	5–15	20–25	27 (23–31)
1. FNA	14.7	38.2			
2. FNA	14.1	21.7			
Risk of neoplasia	30.6	64.7			
1. FNA	26.6	69.1			
2. FNA	38.0	58.7			

**Table 3:** Subclassification of AUS/FLUS cases according to the first and second editions of Bethesda and their risk of malignancy. NOS, atypia not otherwise specified.

Subclassification (First Edition Bethesda)	*n* (%)	Risk of Malignancy	Subclassification (Second Edition Bethesda)	*n* (%)	Risk of Malignancy
Follicular neoplasia	125 (37.8%)	12.0%	Architectural atypia	125 (37.8%)	12.0%
Predominance of Hürthle cells	24 (7.3%)	4.2%	Hürthle cell aspirates	28 (8.5%)	3.6%
Only Hürthle cells	4 (1.2%)	0.0%			
Focal features suggestive of papillary carcinoma	72 (21.8%)	16.7%	Cytological and architectural atypia	72 (21.8%)	16.7%
Cyst-lining cells	7 (2.1%)	28.6%	Cytologic atypia	98 (29.6%)	12.4%
Nuclear enlargement	91 (27.5%)	11.0%			
Atypical lymphoid infiltrate	1 (0.3%)	0.0%	Atypical lymphoid cells	1 (0.3%)	0.0%
Others	7 (2.1%)	14.3%	Atypia NOS	7 (2.1%)	14.3%
